# Methylchloroisothiazolinone/Methylisothiazolinone and Methylisothiazolinone Sensitivity in Hungary

**DOI:** 10.1155/2016/4579071

**Published:** 2016-03-07

**Authors:** Györgyi Pónyai, Ilona Németh, Erzsébet Temesvári

**Affiliations:** Department of Dermatology, Venereology and Dermatooncology, Semmelweis University, Budapest 1085, Hungary

## Abstract

*Background*. Due to allowing of methylisothiazolinone (MI) in cosmetics, cleaning products, and paints, an epidemic of MI-hypersensitivity emerged. Patch testing Kathon CG® (3:1 mixture of methylchloroisothiazolinone and methylisothiazolinone, MCI/MI) does not correctly detect MI contact allergy, due to the low concentration of MI in the test material.* Methods*. A retrospective survey was performed to estimate the prevalence of MCI/MI hypersensitivity in 14693 patients tested consecutively between 1993 and 2014. Moreover, currently 314 patients were prospectively tested with the allergens MCI/MI and with MI during one year.* Results*. MCI/MI hypersensitivity increased retrospectively from 0.5% to 6.0%. By current prospective testing we detected 25 patients (8%) with MCI/MI and/or MI positive reactions. Out of the 25 patients 10 were only MCI/MI positive, 9 were only MI positive, and 6 were MCI/MI and MI positive. If MI had not been tested separately, MI contact allergy would have missed in 36% of all detected cases and in 2.8% of the total 314 patients.* Conclusions*. The frequency of MCI/MI hypersensitivity is increasing also in Hungary. We confirm that, in order to detect MI contact allergy, it needs to be tested separately. A further increase of MI hypersensitivity might be expected in the future as products containing MI are still widely available.

## 1. Introduction

Due to the introduction of Kathon CG (MCI/MI) in the midseventies a worldwide epidemic of contact allergy to it emerged. This preservative became widely used in cosmetics and household cleaning products because of its efficacy. Contact allergy to MCI/MI—mostly provoked by cosmetics—was first reported by de Groot et al. [1]. Frosch and Schulze-Dirks [2] estimated the sensitization rate between 0.4% and 11.1%—with a mean of 3.0%—in their European multicentre study [3] which was similar to our own data (3.4%) observed in the same period [4].

At the beginning of the new millenium MI was allowed as a separate preservative in industrial products (paints, glues) and initially gained attention as an occupational allergen, and it still is nowadays [5]. Due to its use in cosmetics, a new and unprecedented epidemic arose in Europe, in the USA, and in Asia [6–10].

Unfortunately, MCI/MI tested in routine patch test series does not correctly detect MI hypersensitivity, because of the low concentration of MI in the mixture. So, MCI/MI testing in itself did not detect a quite high percent of the MI allergy [9]. Uter et al. already suggested in 2012 the routine separate testing of MI and including it into the standard patch test series [11]. Recently, the recommended MI patch test concentration became 2000 ppm (or 0.2% aqua) [12–14]. We here present our experience with MCI/MI and MI contact allergy and the data in Hungary.

## 2. Material and Methods

(1) We retrospectively reviewed the prevalence of MCI/MI hypersensitivity (European Baseline series* Brial Allergen GmbH, Germany*, chamber:* Curatest*®) in 14693 patients tested consecutively from 1993 until 2014 at the Allergy Outpatient Unit of the Department of Dermatology, Venereology and Dermatooncology of the Semmelweis University.

(2) Moreover, 314 patients were prospectively tested consecutively between February 1st, 2014, and January 30th, 2015, with the standard allergens MCI/MI 0.01% aqua and MI 0.2% aqua (*Chemotechnique Diagnostics, Vellinge, Sweden, *chamber:* IQ Chambers*®). We performed parallel testing with the European Baseline series (*AllergEAZE*®,* Brial Allergen GmbH*,* Germany*, chamber:* Curatest* including the* Brial*® MCI/MI/0.01% aqua/contact allergen), as well. As we had not tested MI separated in routine patch series before, comparative data concerning the past years have not been available.

The occlusion time by testing was 48 h; the allergens were applied on the back. Evaluation of the test was performed at the 60th minute of the occlusion and then on D2, D3, D4, and D7. Reactions were taken as positive 1+ or more intense.

## 3. Results

### 3.1. MCI/MI Hypersensitivity 1993–2014

**Figure 1:**
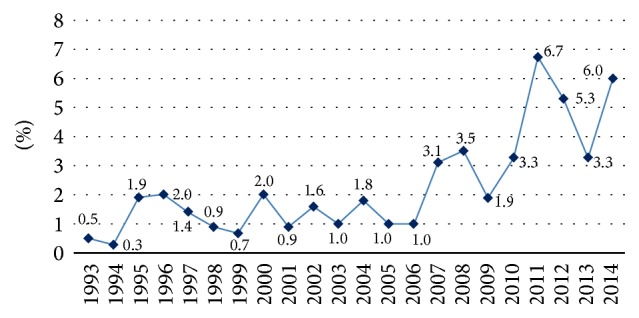
MCI/MI hypersensitivity data in percent (MCI/MI hypersensitive patients/tested patients in the year) at the Allergy Outpatient Unit of the Department of Dermatology, Venereology and Dermatooncology of the Semmelweis University 1993–2014 (*n* = 14693).

Assessing the prevalence of MCI/MI hypersensitivity in our 14693 patients tested consecutively between 1993 and 2014, we detected wavering percent rates (MCI/MI hypersensitive patients/tested patients in the year). Starting from a value of 0.5% (1993: 5/1011 patients), it reached 6.7% (2011: 27/401 patients), 5.3% (2012: 22/413 patients), 3.3% (2013: 13/390 patients), and 6.0% (2014: 23/383 patients) (Table 1, Figure 1).

### 3.2. Testing with MCI/MI (0.01% Aqua) and MI (0.2% Aqua) and Parallel Testing with the Standard* Brial* Baseline Series (Including MCI/MI Allergen) between February 1st, 2014, and January 30th, 2015

The mean age of the 314 tested patients was 48.9 years (range: 13–88 years). There were 79 men with a mean age of 50.6 years (range: 18–88 years) and 235 women with a mean age of 48.4 years (range: 13–88 years).

Regarding the patch test containing MCI/MI, we did not find any differences in the results between the* Brial* and the* Chemotechnique*® allergens.

We detected 25 patients (8%) with MCI/MI and/or MI positivity: 17 women and 8 men. MCI/MI hypersensitivity was detected in 16 cases (5.1%) and MI hypersensitivity in 15 cases (4.8%). Regarding parallel positivities: out of the 25 patients 10 were only MCI/MI positive, 9 were only MI positive, and 6 were concurrently MCI/MI and MI positive.

Thus, MI positivity without MCI/MI positivity was found in 36% of these or in 2.8% of the whole tested population of 314 patients. Among the MI sensitized patients the mean age was 39 years (Tables 2(a), 2(b), and 2(c)). Regarding localisation of contact dermatitis, we observed by MI sensitive patients skin symptoms first of all on the hands, the face, and the scalp. Most typical sources of the allergens were liquid soaps, baths, hair shampoos, hand and face creams, and wet cleansing wipes—mostly rinse-off products.

According to occupational dermatitis, we identified only four patients: two patients (hairdresser and washer-up) with only MI hypersensitivity, one patient (anaesthetist assistant) with both MCI/MI and MI hypersensitivity, and one patient with only MCI/MI hypersensitivity (maid).

Associated contact allergies were detected with, for example, fragrance mix I, fragrance mix II, propylene glycol, nickel sulfate, and paraphenylenediamine (PPD) in 18 patients. There were 3 patients in the MCI/MI and MI sensitive group and 4 patient in the MI sensitive group without associated contact hypersensitivities (Tables 2(a), 2(b), and 2(c)).

## 4. Discussion

In 1987 methylisothiazolinone was considered to be a weak sensitizer in the animal experiments of Bruze et al. [15]. By allowing much higher concentrations use than before an unprecedented allergy epidemic occurred and still occurs worldwide. Among the problematic MI-containing products, cosmetics have been in a leading position since 2005, as the concentration of MI was authorized in both leave-on and rinse-off products up to 100 ppm [16]. Hair care products even proved to be one of the most problematic ones [6, 7, 9].

Apart from the high concentration of MI used, the increase of MI sensitization can also be explained by the fact that the number of cosmetics containing this preservative (baby care products, baths, make-up, hair, nail, skin care, and sun protection products) has doubled in the USA between 2007 and 2010.* Castanedo-Tardana* even nominated MI as the “Allergen of the Year 2013” [7]. MI is in the focus of allergology in our days as well, because of further increasing of contact sensitization, caused by leave-on and by rinse-off cosmetic products [12–17]. The widespread use of MI in several products and cumulative exposures to MI may also be responsible for the high percent of sensitization to it. According to a recent study focusing on contact sensitization in patients with suspected cosmetic intolerance, MI was by far the leading allergen provoking contact sensitization among preservatives [9, 17].

Another recent study examined whether the allowed concentrations of MI in cosmetic rinse-off products have the potential to cause allergic contact dermatitis. According to the results, the rinse-off products with 50 ppm MI or more are not safe for the consumers [18].

In our large study population tested between 1993 and 2014 the prevalence of MCI/MI hypersensitivity gave wavering percent rates, but we detected an increasing rate from the beginning to the endpoint. In this process presumably the MI component of the allergen played an important role [14, 19].

Moreover, we started prospective MI patch testing separately as a routine examination and followed the test results for one year. The 4.8% prevalence of MI hypersensitivity is, though high, may be considered as rather moderate compared to other European data [11, 16–22]. Among the MI sensitized patients the mean age was 39 years. Regarding clinical symptoms, we observed contact dermatitis first of all on the hands, the face, and the scalp [14, 23–25]. The sources of the allergens were mostly rinse-off products (liquid soaps, baths, and hair shampoos).

Interestingly, MI contact allergy without MCI/MI positivity was found in 36% among the patients with positive test reactions to MCI/MI, MCI/MI and MI, and only MI and in 2.8% of the total tested 314 patients. These patients would have been missed if MI had not been tested separately.

In conclusion, MCI/MI and MI contact allergy is a hot topic and an ongoing problem also in Hungary. Despite the restrictions, further increase of MI hypersensitivity may also be expected in the near future as products containing MI are still available widely. The new results worldwide support recommendations for a review of the regulations relating to MCI/MI and/or MI in cosmetics and household products [10, 14, 17, 18].

## Figures and Tables

**Table 1:** MCI/MI hypersensitivity 1993–2014.

Year	Tested patients/year	MCI/MI hypersensitive patients/year	%
1993	1011	5	0.5
1994	636	2	0.3
1995	839	16	1.9
1996	1099	22	2.0
1997	938	13	1.4
1998	802	7	0.9
1999	834	6	0.7
2000	797	16	2.0
2001	799	7	0.9
2002	698	11	1.6
2003	701	7	1
2004	670	12	1.8
2005	637	7	1
2006	612	6	1
2007	538	17	3.1
2008	514	18	3.5
2009	512	10	1.9
2010	480	16	3.3
2011	401	27	6.7
2012	413	22	5.3
2013	390	13	3.3
2014	383	23	6.0

**Table:** (a) MCI/MI hypersensitive patients: localisation of clinical symptoms and associated sensitivities (*n* = 10)

P	Age	Gender	Dg	Localisation of skin symptoms	Patch test hypersensitive reaction		Sources of the allergen	Associated sensitivities
					MCI/MI	MI		
(1)	78	W	ACD	Hands	2+-3+	−	Liquid soap, hand creams	Propolis, Balsam Peru thiuram mix
(2)	70	M	ACD	Trunk, limbs	2+	−	Baths, body lotion	Propylene glycol, quaternium 15, wool alcohol
(3)	58	M	Dysidrosis	Hands	3+	−	Liquid soap	Propylene glycol
(4)	40	W	ACD	Eyelids	2+	−	Hair dye	PPD, nickel sulfate, cobalt chloride
(5)	40	M	ACD	Hands	2+	−	Liquid soap	Fragrance mix II
(6)	27	W	ACD	Neck, scalp	2+	−	Hair shampoo	Nickel sulfate, thiomersal
(7)	68	W	ACD/stasis dermatitis	Legs	2+	−	Wet cleansing wipes	Wool alcohol, wood tar mix, Balsam Peru
(8)	55	M	ACD/stasis dermatitis	Legs	2+	−	Wet cleansing wipes	Propylene glycol, fragrance mix I, tixocortol pivalate
(9)	57	W	ACD	Hands	2+-3+	−	Liquid soap	Nickel sulfate, cobalt chloride
(10)	40	W	ACD	Hands^*∗*^	2+	−	Household cleaners	Nickel sulfate, cobalt chloride

W: woman, M: man, P: patient, Dg: diagnosis, and ACD: allergic contact dermatitis.

^*∗*^Maid, −: negative.

**Table:** (b) MCI/MI and MI hypersensitive patients: localisation of clinical symptoms and associated sensitivities (*n* = 6)

P	Age	Gender	Dg	Localisation of skin symptoms	Patch test hypersensitive reaction		Sources of the allergen	Associated sensitivities
					MCI/MI	MI		
(1)	25	W	ACD	Hands	3+	2+-3+	Wet cleansing wipes, hand creams	−
(2)	17	W	ACD	Hands	2+	1+-2+	Hand creams	−
(3)	56	W	ACD	Hands, legs	2+	2+	Baths, dish washing liquids	Fragrance mix I, propylene glycol
(4)	43	W	ACD	Hands^*∗*^	3+	2+-3+	Liquid soaps	Nickel sulfate
(5)	54	W	ACD	Eyelids	2+	2+	Facial cleansing wipes, hair shampoo	Nickel sulfate
(6)	16	W	ACD	Trunk and limbs	1+-2+	1+-2+	Baths, body lotion	−

W: woman, M: man, P: patient, Dg: diagnosis, and ACD: allergic contact dermatitis.

^*∗*^Anaesthetist assistant, −: negative.

**Table:** (c) MI hypersensitive patients: localisation of clinical symptoms and associated sensitivities (*n* = 9)

P	Age	Gender	Dg	Localisation of skin symptoms	Patch test hypersensitive reaction		Sources of the allergen	Associated sensitivities
					MCI/MI	MI		
(1)	16	W	ACD	Scalp	−	2+	Hair dye	−
(2)	61	M	ACD	Hands	−	2+-3+	Liquid soaps	−
(3)	66	W	ACD	Eyelids	−	2+	Hair shampoo, face creams	Wood tar mix, tixocortol pivalate
(4)	32	M	ACD	Hands	−	2+	Hair shampoo, baths	−
(5)	21	W	ACD	Scalp	−	2+	Hair shampoo, hair dye	PPD
(6)	47	M	ACD	Hands^*∗*^	−	3+	Hair shampoo	PPD, propylene glycol, Balsam Peru, budesonide
(7)	50	W	ACD	Face	−	1+-2+	Face creams	Fragrance mix I, fragrance mix II
(8)	42	W	ACD	Hands^*∗∗*^	−	2+-3+	Dish washing liquids, hand creams	Potassium dichromate, nickel sulfate, wood tar mix, thiuram mix, fragrance mix I, fragrance mix II
(9)	40	M	ACD	Hands	−	2+	Liquid soaps	−

W: woman, M: man, P: patient, Dg: diagnosis, and ACD: allergic contact dermatitis.

^*∗*^Hairdresser, ^*∗∗*^washer-up, and −: negative.
